# Prenatal yoga for young women a mixed methods study of acceptability and benefits

**DOI:** 10.1186/s12884-019-2564-4

**Published:** 2019-11-28

**Authors:** Amanda Styles, Virginia Loftus, Susan Nicolson, Louise Harms

**Affiliations:** 1 Department of Social Work and the Centre for Women’s Mental Health, Royal Women’s Hospital, Melbourne, Victoria Australia; 2 Centre for Women’s Mental Health, Royal Women’s Hospital, Melbourne, Victoria Australia; 3 Department of Social Work, School of Health Sciences, The University of Melbourne, Melbourne, Victoria 3010 Australia

**Keywords:** Adolescence, Pregnancy, Yoga, Trauma, Young women, Benefits, Social connectedness

## Abstract

**Background:**

High rates of psychological-distress, trauma and social complexity are reported among young pregnant women. At the Royal Women’s Hospital, Australia, young pregnant women acknowledge wanting tools to improve maternal wellbeing yet remain challenging to engage in antenatal education and support. While yoga is a widely accepted and participated activity in pregnancy, with demonstrated benefits for adult pregnant women, adolescent women are often excluded from both these yoga interventions and related pregnancy studies.

**Methods:**

This mixed methods study examined the acceptability and benefits of yoga for young women. We recruited 30 participants aged under 24 years, who were offered twice a week, one-hour voluntary prenatal yoga sessions throughout their pregnancy. A medical file audit gathered baseline demographics, pre and post yoga session surveys were administered and brief individual interview were conducted with study participants.

**Results:**

While 26 study participants were positive about the availability of a yoga program, only 15 could attend yoga sessions (mean = 8 sessions, range 1–27). No differences were found in the demographic or psychosocial factors between those who did and did not attend the yoga sessions. The medical file audit found that 60% of all the study participants had a documented history of psychological distress.

Barriers to participation were pragmatic, not attitudinal, based on the timing of the group sessions, transport availability and their own health. All study participants identified perceived benefits, and the yoga participants identified these as improved relaxation and reduction of psychological distress; labour preparation; bonding with their baby in utero; and social connectedness with the yoga group peers.

**Conclusions:**

This study demonstrated yoga was acceptable to young pregnant women. For those who did participate in the sessions, yoga was found to decrease self-reported distress and increase perceived skills to assist with their labour and the birth of their baby. The provision of accessible yoga programs for pregnant young women is recommended.

## Background

Studies show young pregnant women have higher rates of past and current trauma, and of mental health problems than other groups of antenatal women [1–6]. Previous studies also acknowledge the difficulty engaging pregnant adolescents in service use [7, 8], particularly when competing demands and multiple psychosocial issues (for example, depression and abuse) are present [9, 10]. However, with appropriately tailored and complementary support strategies [3, 9, 11, 12], hospitals that specialise in pregnancy and birth care are in a unique and ideal position to engage this hard-to-reach group [13]. Research suggests young pregnant women will attend healthcare during pregnancy [13]; are more likely to receive their first mental health diagnosis in a maternity setting due to their young age [6]; and pregnancy itself can be seen as a window of opportunity for change [9, 14, 15].

Data from The Royal Women’s Hospital (RWH) in Melbourne highlighted that more than half (52%) of maternity patients aged 19 and under-reported living with a mental health condition, and 61% reported a history of depression. Outpatient appointment information revealed only one quarter of these women were seen by a psychiatrist and a half by the dedicated social worker [10]. Therefore, young mothers persistently fall through the gaps, even with assigned and experienced health staff. Clinical experience holds that not all young mothers wish to partake in psychosocial assessments and midwifery care because they do not want to talk about their past difficulties. Some young mothers stated this type of care had a re-traumatising effect. However, they do report wanting tools to address their symptoms.

### Prenatal yoga

Yoga has been practised in the East for thousands of years [16]. Known for its ability to strengthen the social, physical and mental health of women [17], it has emerged as a complementary antenatal practice among pregnant women in the West [18].

In a recent review of the extant literature [19], the surge of yoga research and publications since 2007 has been noted. Yoga has been found to assist with emotional regulation, empowerment to make choices, develop strong connections to others, and enable women to accept and appreciate themselves and their life experiences [20, 21]. In the Australian context, two recent studies have reinforced these findings - with one study of the effectiveness of a prenatal mindfulness group intervention and home-based practice finding that participants also reported improvements in control and confidence in giving birth [22], and another randomized controlled trial showing reductions in depression, anxiety and stress through an antenatal mindfulness program [23].

Furthermore, pregnant women and women with psychological distress may be drawn to prenatal yoga over other group classes offered in their pregnancy [24]. Studies have demonstrated the feasibility of having female trauma survivors participate in yoga groups. For example, in Emerson et al.’s [25] study, after 8 weeks, yoga participants compared to dialectical behavior therapy group participants showed improvements in post-traumatic stress disorder (PTSD) symptoms, commonly associated with depression and other types of anxiety disorders. Similarly, a randomized control trial study has demonstrated not only that PTSD rates decreased for participants after a weekly yoga program, but that compared to control group participants, these benefits were maintained long term [26]. In addition to finding these PTSD symptom reductions, a related qualitative examination also identified self-reported benefits such as enhanced ‘gratitude and compassion, relatedness, acceptance, centeredness, and empowerment’ [20].

Given these findings, prenatal yoga would seem beneficial for adolescents. Yet, the studies outlined above have excluded pregnant women under the age of 18. These studies suggest more research is needed to explore the effects of yoga on women under 25 years of age, who have diverse backgrounds and fewer resources [21, 23]. To our knowledge, this is the first mixed methods study exploring the acceptability and benefits of prenatal yoga for young women aged 24 years and under.

## Method

### Aim

This study aimed to explore the acceptability and perceived benefits of prenatal yoga for young women.

The key objectives of the intervention were to improve the management of psychological distress, and increase feelings of connectedness with other participants and with their unborn baby.

### Health setting and participants

The study was conducted at the RWH in Melbourne, Australia, between 2014 and 2016. The RWH is a large, tertiary maternity hospital with over 7000 births per year and 777 births to mothers under the age of 24 in 2015. The study was approved by the RWH Human Research Ethics Committee (EC00259, Project ID 14/21).

The participants came from the RWH Young Women’s Health Program (YWHP), which provides care for women under 19 years of age, and the mainstream maternity team-care model (for those in this study aged 20–24 years of age) and included both primigravida and multigravida women. All patients attend a routine schedule of visits, with additional visits and referrals for higher risk pregnancies as appropriate. Visits are weekly from 36 weeks. There were 86 births in the YWHP in 2015. At the first visit, all attendees complete a psychiatric screening assessment and a standard adolescent risk assessment during a midwife interview to facilitate appropriate referrals. The YWHP uses a continuity of care model, which includes a specialist midwife coordinator and social worker, along with options of one-to-one child birth education and parent-infant mental health groups and private sessions. Participants in the mainstream maternity team-care model also had the option of one-to-one midwifery support, if appropriate and available.

### Researcher reflexivity

Throughout this project, from the design phase through to data analysis, the researchers engaged in regular critical reflection [27]. Given the capacity to bias the study through our professional involvements and disciplinary lenses – social work, psychiatry and medicine – steps were taken to ensure rigour and trustworthiness measures were in place. For example, in relation to role boundaries, interviewing excluded the involvement of AS, the social worker conducting the yoga group; and throughout the data analysis, triangulation of all coding procedures was undertaken. We were aware that openness to the possibility of finding the intervention was neither acceptable nor beneficial to participants was important.

### Inclusion/exclusion criteria

All adolescents attending the RWH for maternity care are screened by the YWHP coordinator using the HEADSSS psychosocial assessment tool [28]. HEADSSSS is the acronym for the domains of assessment: home; education and employment, eating; activities; drugs and alcohol; sexuality; suicide, depression and self-harm; and safety from injury and violence.

Adolescents deemed to be a mature minor (as legally recognised in Australia as anyone 14 years and over with competency to understand and give informed consent) following assessment were eligible for the study. Exclusion included: women not deemed competent to give informed consent themselves; women older than 24 years; non-English speaking; unsigned or unapproved prenatal exercise screening form; and foetal death in-utero. For health reasons, participants only engaged in the prenatal yoga from 13 weeks’ gestation in line with accredited yoga course recommendations [29].

### Recruitment procedure

Participants were recruited prior to 28 weeks’ gestation, ensuring adequate time [23] to attend a minimum of eight yoga sessions. Recruitment followed the National Health and Medical Research Council [30] guidelines for ethical recruitment of mature minors in research. The study was advertised internally by a flyer, expression of interest form, and by word of mouth from the YWHP midwife, mainstream midwives, and allied health staff. If the young woman was interested in participating, the principal researcher discussed the project in length, a participant information and consent form (PICF) and pre-exercise screening form, assessing safety, were provided, and any further questions answered.

Similar to previous adolescent studies [7, 9, 31], evaluation methods were designed to be completed on the same day as prenatal yoga sessions, antenatal appointments, and if no other option, over the phone. Weekly mobile-phone messages were sent to remind participants of the prenatal yoga session days and times and was a cost-effective way to improve the participation rate. Public transport tickets were offered and afternoon tea was provided.

### Intervention procedure

**Fig. 1:**
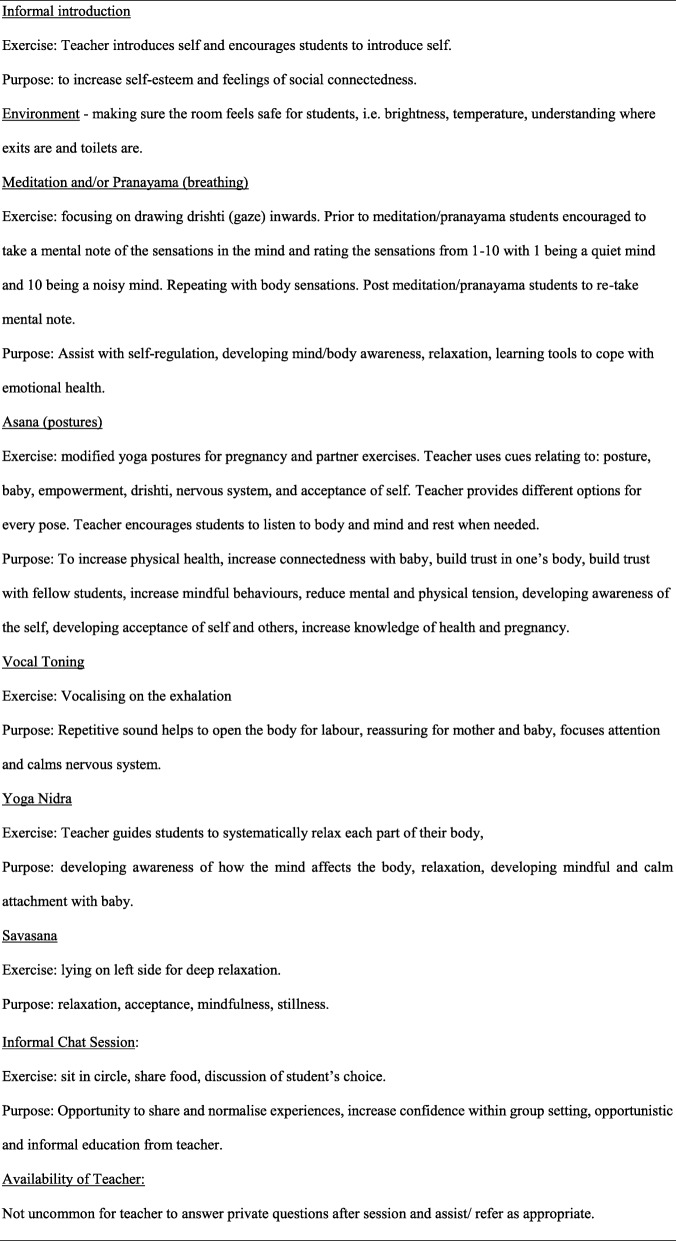
Summary of a Yogabond Session

From 2014 to 2015, the intervention procedure involved 1 hour prenatal-yoga sessions, called Yogabond (Fig. 1).

Sessions were voluntary and consisted of 45 min of postures, breathing, and relaxation exercises, including 15 min of group chat. The intervention was developed and taught by the dedicated YWHP social worker (AS) who was also a trained prenatal yoga teacher. To ensure yoga poses were appropriate for the target population the YWHP social worker sought guidance from a previous YWHP patient who attended prenatal yoga during pregnancy.

Sessions differed from typical prenatal hatha yoga. Mindfulness techniques, used in Muzik, et al.’s [21] study design were similarly used in the Yogabond sessions. References were made to the developing foetus to assist with attachment; breathing exercises, such as ‘nadhi shodhana: alternate nasal breathe’, were used to help anxiety [32]; focusing on the present moment and meditation was used for feelings of depression and anxiety [23]; partner exercises were incorporated to build connections and trust between group participants but also to learn poses for labour (although partners were not participants in the program); emphasising choice and facilitating a safe environment where participants felt safe to approach the yoga teacher, felt safe in their bodies and could begin to trust and listen to their bodies, was paramount [16]. The dual role of social worker and yoga teacher (AS) ensured clinical expertise and ability to respond to concerns of safety. Participants recruited from the YWHP who required ongoing social work support were offered an alternative social worker.

### Design

**Fig. 2:**
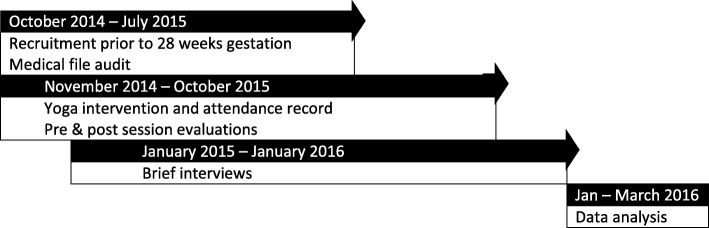
Data collection procedure and timelines

A sequential mixed method design was used [33], which included a medical file audit gathering baseline demographic information; record sheet of sessions attended; pre- and post-session surveys, and a brief individual interview (Fig. 2). The pre- and post-session survey had six questions relating to the perceived psychological benefits, as well as the connectedness to other participants in the session and to the participant’s baby in utero. The survey tool was developed by the research team to specifically evaluate the impacts of the yoga group. Participants rated whether they strongly agreed or disagreed with the questions. The brief interview was semi-structured, with questions relating to five areas: acceptability of yoga, management of psychological distress, connectedness to participants, connectedness to their unborn baby and recommendations.

### Data collection

To ensure data privacy all participants were assigned a code number with paper and electronic data being stored in secure locations. One author (VL), an authorized staff member of the RWH, completed a medical audit file on all 30 participants, in accordance with the ethics approval for this study. Demographic and psychosocial information only relevant to meet the study aims was collected (Table 1).

**Table 1:** Participant demographics from medical file audit

		Yogabond participant		
Medical file audit variable	Responses	Yes (*n* = 15)	No (*n* = 15)	Total number
Country of origin	South America: Argentina	0	1	1
	Australia	9	9	18
	England	0	1	1
	Southeast Asia: India, Thailand	1	1	2
	Africa: Liberia, Sudan	3	2	5
	New Zealand	2	1	3
Level of education	Unknown	3	4	7
	Secondary Years 7–11	3	6	9
	Year 12 commenced or completed	4	2	6
	Tertiary	5	3	8
Primigravida	Yes	8	10	18
	No	7	5	12

The pre- and post-session evaluation was administered, collected, and de-identified by a qualified non-treating social worker, independent from the research team. Prior to each yoga session, the social work colleague was available to meet with all participants - to provide a brief overview of how to complete the survey, advise of its confidentiality and to reduce the likelihood of wanting to please the yoga teacher with their answer [34].

The brief interviews were conducted in the RWH social work department or over the phone, administered by VL, not yet known to participants. The approximate time for interview was scheduled at 35 week’s gestation. This allowed time for rescheduling failed-to-attend appointments, and secondly, the timing avoided the possible influence of hormone influenced mood changes in the early postpartum stage known as the ‘baby blues’. Data are unclear regarding their influence [35] however, it is estimated that 60–80% of all new mothers experience postnatal blues [5, 36], and may skew an interview response. When completed face-to-face, the interview was audiotaped. Participants who spoke about psychological distress were offered follow-up support with an appropriate professional. The interviews were typically five to 10 min.

### Data analysis

Demographics and psychosocial features were documented. Attendance rates demonstrated acceptability of the program as did responses in the brief interview. The raw data of the pre- and post-session surveys were analysed using descriptive statistics, primarily the mode or most frequent response. For each question, the most frequent responses were compared against participant’s first and tenth yoga session. This provided data relating to longitudinal benefits of a yoga practice. Given the small sample size, inferential statistics were not used.

Given the brevity of the interviews, a content analysis [37] was conducted with the data, rather than an in-depth thematic analysis [38]. The content analysis was undertaken by three of the researchers (AS, VL & LH) through a consensus process. Responses to the interview questions were coded and a frequency analysis was conducted of these codes. Participants who attended the yoga sessions are distinguished by letters ‘YP’ and the number assigned to them in the study. Participants who did not participate in yoga are distinguished by the letter ‘P’ and study number, for example, ‘P5’. However, given the small sample, along with the finding from the medical file audit that yoga participants did not differ on demographic or psychosocial characteristics from non-participants, the content analysis of interview data relating to psychological distress did not include a comparison of the yoga participants and non-participants. Instead, the focus was on understanding this sample of young pregnant women as a whole group.

### Sample

The number of participants for each data collection method were: medical file audit (*n* = 30); attendance record (*n* = 16); pre- and post-session evaluations: session 1 (*n* = 16) and session 10 (*n* = 6); and final brief interview (*n* = 28). The number of participants varied in each data collection method due to no participation in the yoga sessions or relocation of address.

## Results

The study recruited its target of 30 participants, with 53 % recruited from the YWHP and the others recruited from the mainstream maternity clinics at the RWH. With the inclusion age being up to 24 years, ages ranged from 14 to 24 (mean age 20 years). The results are presented under the following five headings.

### Demographic characteristics and psychosocial risk factors of participants

The demographic characteristics of the 30 participants are presented in Table 1. Although most participants were born in Australia, the other 40% were from six different countries of origin, reflecting the cultural diversity of young women at the RWH.

As shown in Table 2, there were no significant differences between yoga participants and those who did not attend in relation to their psychosocial characteristics. All but two study participants were noted as being in a relationship, although of these, 3 yoga participants and 3 non-participants were noted as being in ‘on and off’ relationships.

**Table 2:** Frequency of psychosocial characteristics by yoga group participation

	Yogabond participant		
Medical file audit variable	Yes	No	Total number
In a relationship	15	13	28
Current family violence	1	1	2
History of family violence	5	6	11
Current partner violence	3	3	6
History of partner violence	3	4	7
Current substance abuse issues	0	1	1
Past substance abuse issues	2	5	7
Current mental health issues	9	9	18
Past mental health issues	6	7	13
Current child protection involvement	0	2	2
History of child protection	3	4	7

The most common area of psychosocial complexity for all the participants was in relation to current and past mental health problems. Those reporting histories of family and partner violence, and substance abuse, were a small sub-sample experiencing these multiple factors.

### The acceptability of Yogabond

While most study participants (*n* = 26) found the idea of the yoga program during their pregnancy highly acceptable and worthy of recommendation, only 15 (50%) were able to engage in it. These 15 young women participated in one or more yoga sessions (mean = 8.9 sessions, range 1–27) throughout the duration of the study.

#### Reasons for non-attendance

The reasons for non-attendance, described by 17 yoga and non-yoga participants, were all external factors to the intervention. The most common reasons given for non-attendance related to the scheduling and accessibility of the group; the participant’s own ill health or physical limitations; competing demands; and transport issues.

#### Reasons for attendance

For those able to overcome these barriers, three key motivations for attendance were identified. Fifteen of the yoga participants reported both relaxation and broader physical benefits as the two most commonly stated reasons for wanting to attend, reflected in these two comments:

“I’m scared. I do yoga to be relaxed” (YP5)

“To get in touch with my body” (YP11).

The third most commonly stated reason was to support birth preparation and wellbeing in pregnancy for the mother and baby. As two young women said:

“I heard it makes it easier to labour because you are flexible” (YP3)

and

“Good for me and baby in pregnancy” (YP13).

An important dimension of the acceptability of the sessions for those who did attend was the style of the class. Five participants reported that the most likeable aspect was the style of the class. Yoga participants identified these aspects, for example:

“Sitting down after and having a talk with (Yoga teacher) and the other young women” (YP13)

“It was not a formal routine … There was space to ask for help” (YP28)

“You go in upset, by the time you finish it’s the opposite feeling” (YP26).

Positive participant feelings were also expressed regarding the qualities of the prenatal yoga teacher. For example:

“Teacher was great: hard to make young girls feel comfortable and she helped me feel very comfortable” (YP11)

“Teacher very good, kind, generous. Recommend she keep doing it” (YP16).

### The perceived benefits of participating in Yogabond

The perceived benefits of participating in Yogabond were examined both through the pre and post evaluation surveys with the yoga participants, and through the interviews with all study participants.

Table 3 shows the survey responses of yoga participants pre and post their sessions, comparing sessions one to ten. Notwithstanding the overall small number of participants, particularly for those attending ten sessions, the trend observed across all areas was positive – both from the pre to post evaluation for session 1, and over time, from session 1 to session 10. Noteworthy was the exception in relation to reported relaxation levels – with the small number of women attending session 10 reporting lower relaxation levels than session 1. However, the post-session reporting showed an increase in reported relaxation levels, indicative of a positive impact of the session itself.

**Table 3:** Pre-and-post yoga session evaluations

Question	Time	Strongly Agree	Somewhat agree	Undecided	Somewhat disagree	Strongly disagree
1. I feel relaxed now	Pre-session 1 (*n* = 16)	37.5%	56.25%	0	6.25%	0
	Post Session 1 (*n* = 16)	87.25%	12.5%	0	0	0
	Pre-Session 10 (*n* = 6)	33.3%	16.6%	33.3%	16.6%	0
	Post session 10 (*n* = 6)	66.6%	33.3%	0	0	0
2. I feel good about being me now	Pre-session 1 (*n* = 16)	50%	50%	0	0	0
	Post Session 1 (*n* = 16)	100%	0	0	0	0
	Pre-Session 10 (*n* = 6)	33.3%	33.3%	33.3%	0	0
	Post session 10 (*n* = 6)	50.0%	50.0%	0	0	0
3. I feel comfortable now around the other young women in the Yogabond session	Pre-session 1 (*n* = 16)	12.5%	25%	62.5%	0	0
	Post Session 1 (*n* = 16)	62.5%	31.25%	6.25%	0	0
	Pre-Session 10 (*n* = 6)	66.6%	33.3%	0	0	0
	Post session 10 (*n* = 6)	83.3%	16.6%	0	0	0
4. I feel accepted now by the other young women in the Yogabond session	Pre-session 1 (*n* = 16)	18.75%	18.75%	62.5%	0	0
	Post Session 1 (*n* = 16)	75.0%	0	25.0%	0	0
	Pre-Session 10 (*n* = 6)	83.3%	0	16.6%	0	0
	Post session 10 (*n* = 6)	66.6%	16.6%	16.6%	0	0
5. I feel confident now to talk to other young women in the Yogabond session	Pre-session 1 (*n* = 16)	18.75%	43.75%	37.5%	0	0
	Post Session 1 (*n* = 16)	62.5%	25.0%	12.5%	0	0
	Pre-Session 10 (*n* = 6)	66.6%	16.6%	16.6%	0	0
	Post session 10 (*n* = 6)	83.3%	16.6%	0	0	0
6. I often feel connected to my baby	Pre-session 1 (*n* = 16)	81.25%	12.5%	6.25%	0	0
	Post Session 1 (*n* = 16)	87.5%	6.25%	6.25%	87.5%	6.25%
	Pre-Session 10 (*n* = 6)	83.3%	16.6%	0	0	0
	Post session 10 (*n* = 6)	100%	0	0	0	0

The interviews provided further insights into these perceived benefits of the yoga sessions, across four key areas.

#### Relaxation and psychological benefits

All fifteen of the yoga participants interviewed stated the sessions helped them feel better psychologically. When asked to describe this feeling after a session, participants spoke of feeling relaxed, including positive emotional responses and physical bodily responses. For example:

“After session, I came out happy and relaxed” (YP13)

“Felt better physically” (YP28).

The psychological benefits included learning tools to manage psychological distress; for example:

“Emotions are not us, they will pass … when we feel upset, take it as it comes this helps when you feel down” (YP26)

“Helped me learn how to control self … Sometimes I worry about little things – this helped me not to” (YP13).

All of the yoga participants stated that they had practised prenatal yoga on their own.

#### Opportunity to bond with their unborn baby

As noted in Table 3, 12 of the 15 (80%) yoga participants agreed that Yogabond sessions helped them feel connected to their baby. They also spoke about these benefits in the interview, identifying ways in which they were newly attuned to their baby. For example, they spoke of:

“ … spending time with baby and hugging baby” (YP16)

“Sense of being calm and listening to the baby” (YP26).

For one participant in a first session of yoga, there seemed to be a shift from often feeling connected to their baby to then disagreeing with this statement, a finding that is complex to interpret but potentially indicative of new insight.

#### Benefits for labour

Eight of the 15 (53.33%) yoga participants perceived the benefits for labour as the most important benefit. They reported feeling more prepared for labour and parenthood. They also saw that the yoga training gave them the opportunity to learn positions and breathing techniques in readiness for labour, including their rehearsal of specific exercises. Many stated that the yoga training gave them insight into the ability to be calm in labour, as illustrated in these two comments:

“Overcoming stress and calming self is good for labour” (YP21)

“Breathing is likely to help calm self so I don’t depend on others too much” (YP24).

#### New social opportunities and connections

As noted earlier in Table 3, the yoga participants’ social connectedness extended with their peers over the duration of the Yogabond sessions. The opportunity to make real social connections and friendships was seen to be of almost equal importance to the perceived benefits of yoga for their labour. Seven of the 15 (46.7%) yoga participants interviewed described positive changes in their connections with other young women. For example,

“The only thing I do is go to class … If it wasn’t for that I wouldn’t have met the girls” (YP26).

Thirteen of these fifteen yoga participants reported in the brief interview that other participants in the yoga sessions helped them feel good.

### Understanding the psychological distress of young pregnant women

In order to understand the lived experience of psychological distress among young pregnant women, an interview question focused on whether all study participants ‘had significant stress, anxiety, or worry’ later in their pregnancy. Sixteen of the 26 respondents (62%) to this question stated that they did. Of these, 14 yoga and non-yoga participants identified the probable cause of their distress, summarized in Table 4.

**Table 4:** Identified causes of psychological distress in pregnancy

	Number of respondents (*n* = 14)
Lack of family support and understanding • Lack of practical support from family • Family disapproval • Having to move back in with family of origin for support was stressful	4
Cultural issues • Cultural shame. • Cultural reasons (dowry)	4
Concerns about the baby • Not feeling pregnant due to small size of belly • Worry about baby • Concerned about small size of baby in utero and struggling with complex medical terminology • Concern about impact of stress on foetus	4
Mental health gPartner’s financial and legal issues impacting on mental health • History of anxiety in previous pregnancy • Pre-existing mental health condition	3
Relationship with father of the baby • Relationship difficulties – no support, poor communication, no affection from partner • Relationship issues with Father of baby • Relationship stress with Father of baby	3
Not knowing what to expect (pregnancy and emotional changes) • Because it’s my first baby • Physical changes	3
Intimate partner violence	3
Employment • Concern of employment security • Workforce discrimination • Stress of working and being pregnant	3
Education pressures • Ceasing education • Managing study	3

As Table 4 shows, the reasons for this psychological distress varied, demonstrating the competing biological and social challenges of young mothers. Young women spoke of their complex relationship circumstances with partners and wider family support, including cultural concerns. The severity of these concerns was reflected in this young woman’s statement:

“Fearful of violence from father and step-mother and father of baby” (YP5).

For others, the psychological distress related to coping with the mental health aspects of pregnancy – concerns about the baby, in particular.

“ … being pregnant for the first time is very stressful because of the emotional changes” (YP3).

While the psychological distress was primarily related to their partner, unborn baby and extended family and cultural supports, it is important to note that these young women were also struggling with worry about their future employment and education prospects.

## Discussion

With an understanding that pregnant young women need and want tools to reduce symptoms of antenatal anxiety and depression, this study offered an alternative to the mainstream talk-based therapies, in a maternity setting. Using prenatal group yoga sessions tailored to young women’s needs, the study explored prenatal yoga’s acceptability and benefits for these young pregnant women.

Acceptability of the study was measured by the number of Yogabond sessions attended, and by the positive responses from the brief interview. In terms of acceptability, while the majority of the study participants found the concept of participating in a yoga program in their pregnancy positive, only half of the sample were able to access it. The style of the Yogabond sessions was perceived by participants as the most likeable aspect. Participants’ emphasised the yoga teacher’s ability to provide choice and communication [16], enabling an environment of emotional safety [39], tailored to participants’ needs [11]. Importantly, consistent with previous studies, this increased participation in antenatal care [7–9].

Given that the barriers to participation were found to be pragmatic in nature, not attitudinal, we conclude that the program was acceptable to participants. For future programs to be successful, addressing these pragmatic barriers is important – providing sessions at accessible times, in particular, which can be complex in the context of the hospital scheduling of outpatient appointments. Young women were unable to resource additional transport to attend the hospital on non-clinic days, reflective of their often very limited financial resources.

This finding of acceptability is significant in the context of the high levels of stress and trauma many of the study participants were experiencing. Our study replicated previous findings of psychological distress at the RWH [10], with nearly two thirds of all the interview participants reporting significant psychological distress in their pregnancy. Consistent with previous studies [1, 2], cultural and trans-generational complexity, lack of family support, the unknowns of first-time pregnancy, intimate partner violence, and educational and employment issues specific to the outcome of pregnancy and youth, were reported as common issues leading to symptoms of anxiety and depression in pregnancy. Participating in the yoga group gave the opportunity for the young women to discuss some of these concerns. However, it is important to note that these concerns may have been why other young women were reluctant or unable to join a group, despite their expressed interest in doing so.

Critically, the yoga sessions did appear to support the management of this psychological distress, seen as a key benefit. All interview respondents who participated in the yoga sessions reported feeling psychologically better even after one session. With the mean number of eight sessions of participation, our findings are consistent with other studies that have shown that the symptoms of stress can reduce after 8 weeks of regular practice [25]. A finding that may be inconsistent with previous research findings [20, 26] was the reporting of lower relaxation levels prior to the tenth yoga session. However, given the post-session relaxation levels were improved, this may have been due to increased stress levels later in the pregnancy, or increased insight into and reporting of their own relaxation state.

Another key finding of the study was the perceived benefit for preparation of labour. Whilst Bryne et al.’s [22] mindfulness-childbirth study suggests further trials to emphasise and explore barriers to a home practice, our study participants who attended the Yogabond sessions voluntarily practised at home, increasing self-efficacy and reinforcing the potential benefits for labour and wellbeing.

The third key benefit was the opportunity for participants to connect with others, be that their peers in the group, or perhaps more importantly, their own baby in utero. This yoga program provided a formal opportunity to engage these young women in social connections, which in turn they saw as enhancing their own parenting connections. This is a key finding, given how at risk this group of young women is typically perceived to be in engaging in their own health and mental health support [21–23].

### Limitations of the study

The funding and time frames for the project limited the scope of this study. As a result, one of the major limitations of this study was its small, self-selected and voluntary sample. Another limitation was the variable attendance rates of the young women at the yoga sessions, and subsequent variation in the responses to the data collection tools used in this study. While retention methods, such as weekly mobile phone updates and public transport tickets were given, and were perceived as helpful, it is unknown as to whether they increased participation. These variations make generalisability of the findings limited.

The third limitation of the study is knowing whether the yoga intervention itself was the most beneficial in addressing the depression and anxiety symptoms or if other factors, such as social inclusion, teacher qualities, or ‘prenatal’ yoga with a focus on labour preparation, may account for the findings. Or it may have been the young women’s intention for change - that is, feeling relaxed, increasing their sense of community, and desire to practise at home – that affected the study outcomes rather than the yoga intervention itself. This study may demonstrate that young pregnant women who are drawn to mind-body practices will benefit the most.

Further large scale and longitudinal research is needed to enable all these dimensions to be systematically examined. Other barriers and enablers to participation, such as the young women’s engagement in their prenatal care at the hospital, the benefits of continuity of midwifery care provided, and the pragmatic factors described earlier, would all need to be included in this future research.

Notwithstanding these limitations, the study has provided insights into how a typically difficult to engage group of young women were positive about the potential for a targeted yoga program, and for those who could attend, positive about the benefits they received from doing so.

## Conclusion

This article has explored the benefits of prenatal yoga for young pregnant women in a busy tertiary maternity hospital setting. It has demonstrated that prenatal yoga was acceptable and beneficial for many of these young women. It is critical that clinicians and educators recognise the significant self-identified stressors and trauma amongst these women, who are often reluctant to engage in mainstream psychosocial interventions. Nevertheless, with high rates of psychological distress, many of these women were prepared to attend prenatal yoga. For those who did, it built on opportunities to prepare for labour and parental bonding, and increased confidence and social connectedness, when practising with other young women in similar situations.

## Data Availability

The datasets generated and/or analysed during the current study are not publicly available due to the small primarily qualitative nature of the study, but are available from the corresponding author on reasonable request.

## Abbreviations

HEADSSS: Psychosocial assessment tool measuring: home; education and employment, eating; activities; drugs and alcohol; sexuality; suicide, depression and self-harm; and safety from injury and violence
PICF: Participant Information and Consent Form
PTSD: Posttraumatic Stress Disorder
RWH: Royal Womens Hospital
YWHP: Young Women’s Health Program
